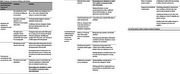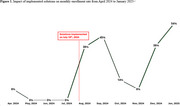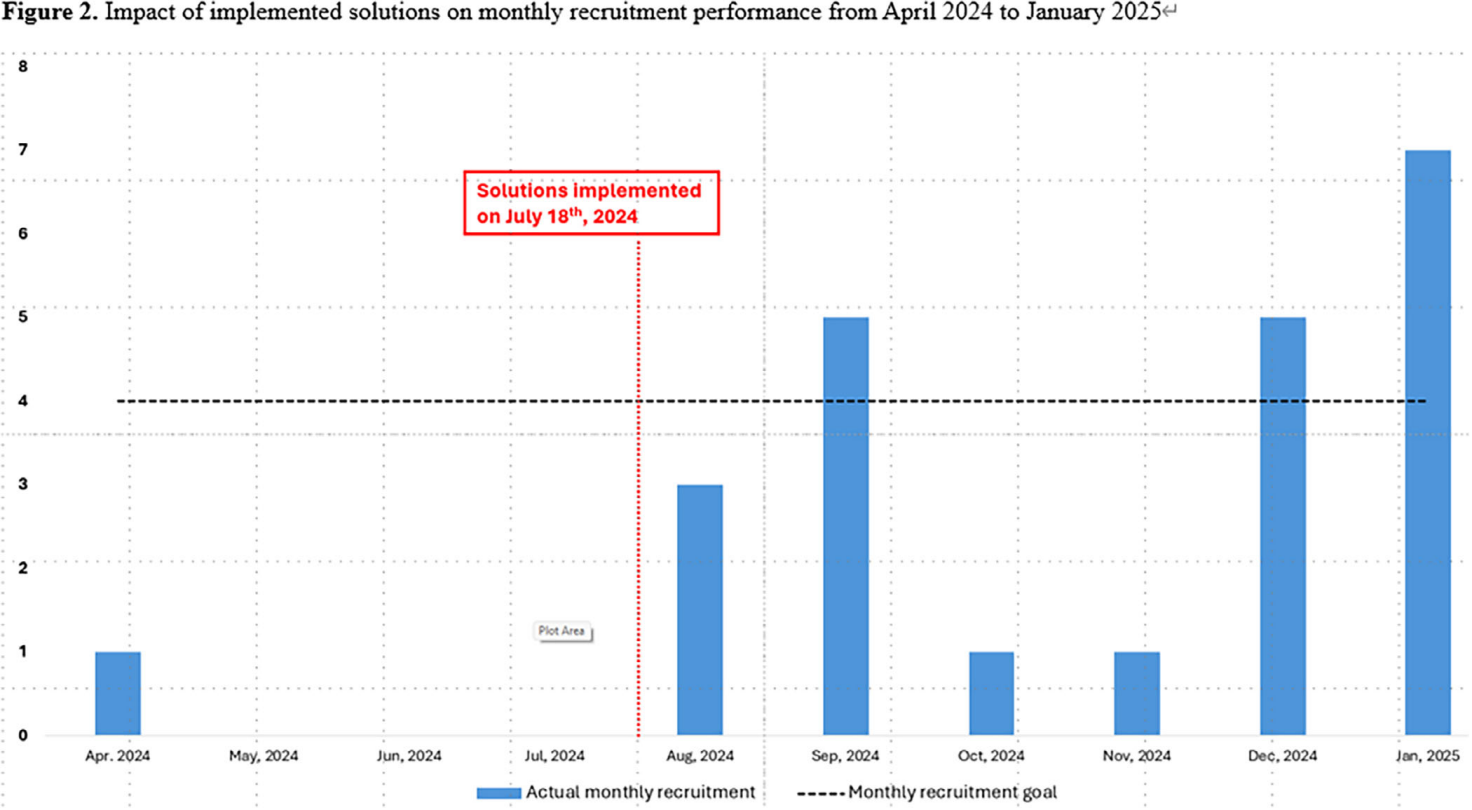# Recruiting care partners of hospitalized people living with dementia: insights from a pilot randomized controlled trial

**DOI:** 10.1002/alz70858_101098

**Published:** 2025-12-25

**Authors:** Te‐Lien Ku, Kayla Dillon, Henry P Karelitz, Jenna Sanders, Anne M Mortensen, Shelly C VanDenbergh, Emmanuel Quarcoo, Dani M Edwards, Courtney Harvey, Michele L Shoemaker, Beth E Fields

**Affiliations:** ^1^ School of Pharmacy, University of Wisconsin‐Madison, Madison, WI, USA; ^2^ School of Education, University of Wisconsin‐Madison, Madison, WI, USA; ^3^ University Hospital, UW Health, Madison, WI, USA

## Abstract

**Background:**

Care partners (CPs) are vital to supporting people living with dementia (PLWD) before, during and after hospitalization. Recruiting CPs for research is critical to developing effective, real‐world interventions, yet evidence from hospital settings remains scarce. This study highlights the challenges and lessons learned from ten months of recruiting CPs of PLWD for a hospital‐based pilot trial.

**Method:**

From April 2024 to January 2025, the study team conducted nine virtual meetings with hospital nurse champions and coordinators and five in‐person study team meetings to discuss study updates, recruitment challenges, and solutions. All meetings were video recorded, transcribed verbatim, and analyzed thematically using the Social Marketing Framework. Two researchers independently coded the data, with a third resolving discrepancies to achieve consensus. Data triangulation and member checking with nurse champions and coordinators ensured analytic rigor.

**Result:**

Our preliminary results identified 13 recruitment challenges across three domains (Table 1): identifying CPs, promoting and advertising the study, and communicating and collaborating as an academic‐health system team. To address these challenges, we implemented 20 solutions. The study team attended monthly hospital electronic health record office hours to improve familiarity with the patient chart system and took over screening tasks to reduce nurse champions’ time demand. Nurse champions were encouraged to contact CPs more frequently through in‐person check‐ins and phone calls, supporting CPs’ participation. Increasing study poster/flyer visibility, sharing study information during unit check‐ins and in‐service meetings, and offering incentives enhanced staff awareness and trust. Standardizing communication channels improved the study team's responsiveness to issues, minimized miscommunication, and reduced redundancy. As a result, the average enrollment rate surged to 33.4% between August 2024 and late‐January 2025, up from 1.9% between April and July 2024 (Figure 1). The monthly recruitment goal was met and exceeded in three of the six months, compared to none before implementation (Figure 2).

**Conclusion:**

Recruiting CPs in hospital settings is challenging, necessitating close collaboration with clinicians to navigate logistical and procedural complexities. Lessons learned from our study highlight challenges and implemented solutions led to a marked improvement in recruitment outcomes, offering valuable insights to enhance CP recruitment for research in similar environments.